# Reconstruction with Total Scapular Reverse Total Shoulder Endoprosthesis after Radical Tumor Excision

**DOI:** 10.1155/2021/1968621

**Published:** 2021-01-25

**Authors:** Michael J. Harvey, Howard G. Rosenthal

**Affiliations:** ^1^University of Missouri Kansas City School of Medicine, Kansas City, MO, USA; ^2^University of Kansas, Sarcoma Center, Overland Park, KS, USA

## Abstract

Malignant musculoskeletal tumors about the shoulder girdle region involving the scapula are fairly rare, but when diagnosed, challenging and complex surgical treatment may be warranted with the primary goal of improving patient survival. These tumors are typically extensive and infiltrative at the time of presentation, requiring radical resection to achieve margins and obtain local tumor control. Historically, forequarter amputation or flail extremity were the mainstays of treatment in these cases. Presently, with recent advances in diagnostics, imaging, adjuvant therapies, and surgical treatment, many patients presenting with malignant tumors involving the scapula are candidates for limb salvage surgery. Reconstruction with endoprosthesis seems to have gained acceptance as the preferred surgical treatment for such lesions, as this intervention has resulted in improved postoperative function and cosmesis, with an acceptable complication rate. We present our experience with recent advancement in these surgical efforts in the form of shoulder girdle reconstruction with total scapular reverse total shoulder prosthesis after radical tumor excision.

## 1. Introduction

Chondrosarcoma, although a rare malignancy overall, comprises approximately 30% of cancers involving the skeletal system. When these lesions originate about the shoulder girdle region, by the time of presentation they are usually fairly extensive and can involve the rotator cuff and surrounding musculature, glenohumeral joint, and the proximal humerus [1–6]. Unfortunately, this tumor has been found to respond poorly to chemotherapy and radiation therapies, leaving surgical resection as the recommended treatment. Historically, definitive treatment of large chondrosarcomas involving the shoulder girdle region involved flail extremity or forequarter amputation. The results of these procedures could be described as minimally functional and nonaesthetic. More recently, improved outcomes have been reported after scapular reconstruction with prostheses or allograft [7]. Pritsch et al. retrospectively compared outcomes of humeral suspension versus endoprosthetic reconstruction after total scapulectomy and found significantly improved functional and cosmetic results with reconstruction. They recommended that reconstructive efforts be undertaken when feasible [8].

In this manuscript, we describe a surgical technique and discuss a series of three patients in whom a single surgeon (HGR) performed reconstruction with total scapular reverse total shoulder prosthesis after radical excision of chondrosarcoma about the shoulder girdle.

## 2. Cases

### 2.1. Case 1

**Figure 1:**
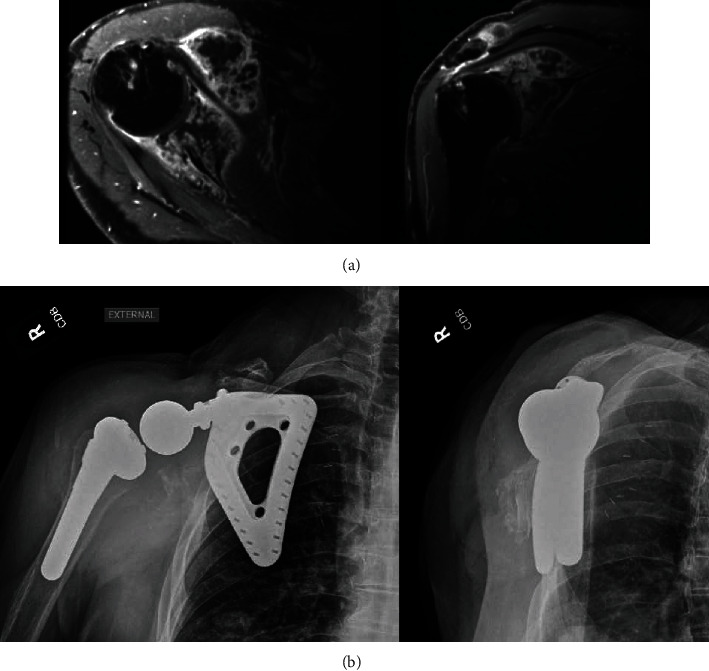
(a) MRI demonstrating expansive, infiltrative soft tissue malignancy about the scapula and shoulder girdle. (b) Eight-month postoperative radiographs demonstrating well-fixed implants in appropriate alignment.

This is a 65-year-old gentleman who was referred from his primary care provider for further evaluation of a right shoulder lesion that was found during routine work-up of atraumatic shoulder pain. After performing appropriate imaging studies and open biopsy, he was diagnosed with a large Grade II chondrosarcoma involving the right scapula and glenohumeral joint (Figure 1(a)). The decision was made to proceed with radical resection of the tumor and reconstruction with total scapular reverse total shoulder replacement prosthesis.

Postoperatively, this patient's recovery was complicated by persistent wound drainage and prosthetic shoulder dislocation that occurred in physical therapy about six weeks postoperatively. He was brought back to the OR at that time for I&D and open reduction of his shoulder dislocation with antibiotic bead placement. Due to persistent infection, he subsequently underwent repeat irrigation and debridement with removal of deep implants, resterilization and single-stage reimplantation of the custom-made prostheses. This was a final attempt to eradicate infection while retaining the custom made implants. This surgery was performed about 8 weeks after the index reconstruction. During reimplantation, the soft tissue repair about the prosthetic shoulder joint was augmented with semitendinosus allograft.

Subsequently, he progressed well, and by about 8 months postoperatively, he had 5/5 muscle strength about the shoulder and was satisfied with his functional progress and cosmetic outcome (Figure 1(b)).

Unfortunately, about one year postoperatively, he was diagnosed with chondrosarcoma recurrence and evidence of pulmonary metastasis. He eventually underwent forequarter amputation.

### 2.2. Case 2

**Figure 2:**
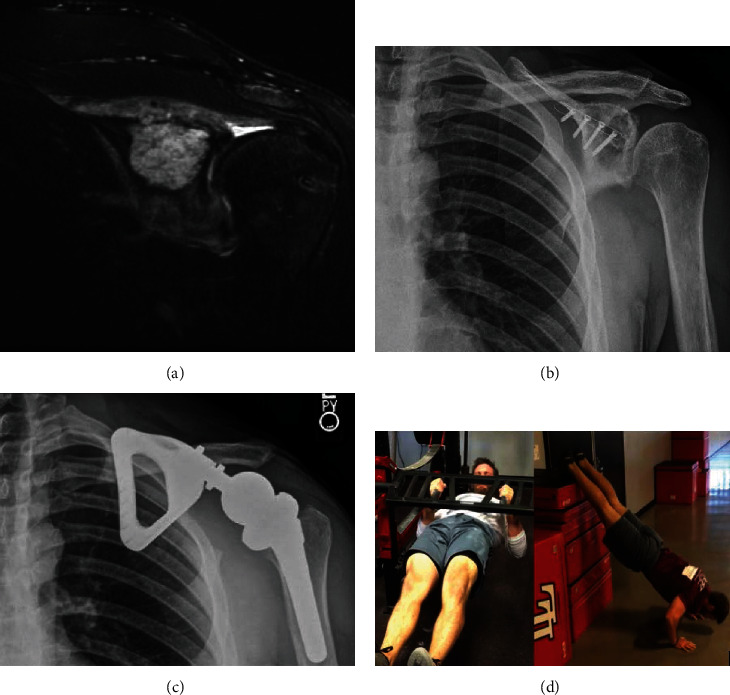
(a) MRI demonstrating malignant lesion of the scapula involving the subglenoid region of the shoulder. (b) Shoulder radiograph after index surgery which involved intralesional curettage, cryosurgery, grafting, and prophylactic internal fixation of the left glenoid. (c) Three-year postoperative radiograph after shoulder reconstruction. (d) Patient performing cross-fit activities postoperatively.

This is a 31-year-old male who was referred from his primary care provider for further evaluation of a left shoulder lesion that was found during work-up of atraumatic shoulder pain. After performing appropriate imaging studies, and after formal biopsy, he was diagnosed with low-grade chondrosarcoma with significant involvement of the glenohumeral joint (Figure 2(a)). This patient's index surgery involved intralesional curettage, cryosurgery, grafting, and prophylactic internal fixation of the left glenoid (Figure 2(b)). The patient did well initially, but unfortunately, local recurrence was confirmed with surveillance MRI and biopsy about 9 months after his initial procedure. At this time, his tumor was found to involve a significant portion of the left glenoid with extension into the scapular body. The decision was made to proceed with radical resection of the tumor and reconstruction with total scapular reverse total shoulder replacement prosthesis (Figure 2(c)).

At his first postoperative visit 2 weeks after surgery, the patient was doing well, no longer taking narcotic pain medications, and compliant with full-time shoulder immobilizer use. He was seen one month after surgery and was started on a short course of oral antibiotics for the treatment of peri-incisional cellulitis contributing to delayed wound healing. Although wound healing progressed slowly, his superficial infection resolved and his incision eventually healed. The patient was able to resume range of motion and strengthening therapy with physical therapy about 8 weeks postoperatively. At the four-month postoperative visit, his only complaint was occasional peri-incisional numbness. He had returned to weightlifting and was already able to perform modified push-ups, pull-ups, squats, and deadlift exercises (Figure 2(d)). One year postoperatively he had about 80 degrees of active forward elevation of the shoulder and 5/5 strength with shoulder abduction and external and internal rotation. Most recently, he was seen 3 years post-operatively and did not have any evidence of local recurrence. He had continued powerlifting activities and was very pleased with the functional and aesthetic outcome of his surgery.

### 2.3. Case 3

**Figure 3:**
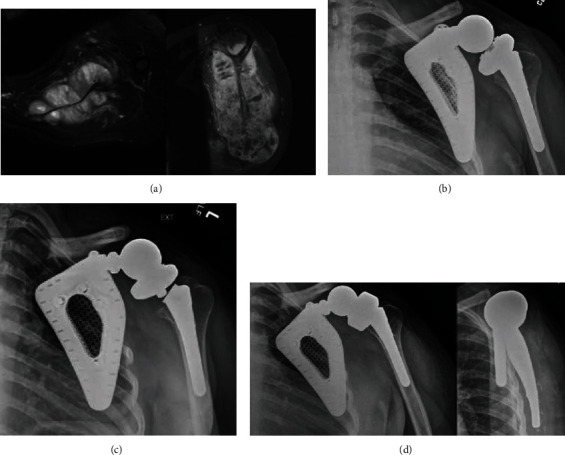
(a) MRI demonstrating expansive, infiltrative soft tissue malignancy about the scapula. (b) Immediate postoperative radiograph. (c) Radiograph demonstrating dissociation of the humeral tray and stem at the Morse taper six months postoperatively. (d) Immediate postoperative radiographs after revision of the humeral component.

This is a 44-year-old male who was referred from his primary care provider for further evaluation of a left shoulder lesion that was found during work-up of atraumatic shoulder pain. After performing appropriate imaging studies and biopsy, he was diagnosed with high-grade chondrosarcoma involving the left scapula (Figure 3(a)). The decision was made to proceed with radical resection of the tumor and reconstruction with total scapular reverse total shoulder replacement prosthesis (Figure 3(b)).

Postoperatively, this patient's acute recovery from surgery was unremarkable; at four weeks, he was allowed to discontinue shoulder immobilization and begin physical therapy for range of motion. By his six-week postoperative visit, he had begun strengthening and was allowed to perform activities as tolerated with his left shoulder. He was progressing well until about 6 months postoperatively when he experienced sudden loss of range of motion and mechanical symptoms in the shoulder. Radiographs were obtained which demonstrated dissociation of the morse taper connecting the humeral tray to the stem (Figure 3(c)). He was brought back to the operating room for revision of his humeral component, which consisted of exchange to a larger tray with a retentive polyethylene (Figure 3(d)). At six weeks postrevision surgery, this patient was participating well in therapy and able to perform activities of daily living without significant functional impairment. Most recently, he was seen about 2.5 years after his index reconstructive surgery and was satisfied with his functional and aesthetic outcomes.

## 3. Surgical Technique

A long incision is made over the anterior aspect of the shoulder above the axillary fold and continued above the top of the shoulder to the posterior side and extended down to the most inferior tip of the scapula. If appropriate, any previous biopsy tracts are to be excised. Deep dissection involves radical tumor excision, taking care to obtain clear margins and avoid contamination. Great care is taken to avoid the lung apex, and to avoid violation of the brachial vessels and brachial plexus within the axillary region. Superiorly, circumferential subperiosteal dissection of the clavicle is performed to expose its lateral third. The lateral third of the clavicle is then osteotomized. Dissection is carried down over the posterior aspect of the scapula, elevating a portion of the supraspinatus and trapezius muscles. Care is taken to maintain the muscles in wide en bloc fashion along the superior aspect of the scapula. At the scapular spine, subperiosteal dissection is carried out to the medial border of the scapula. At this point, the inferior half of the scapula can be identified and the infraspinatus muscle elevated from its origin. The medial scapular border musculature is maintained if possible, to aid in later reconstruction and soft tissue repair. Subperiosteal dissection is then completed to the most inferior and lateral margins of the scapula, exposing entire lateral border, once again taking care to maintain muscle insertions to aid in soft tissue repair.

**Figure 4:**
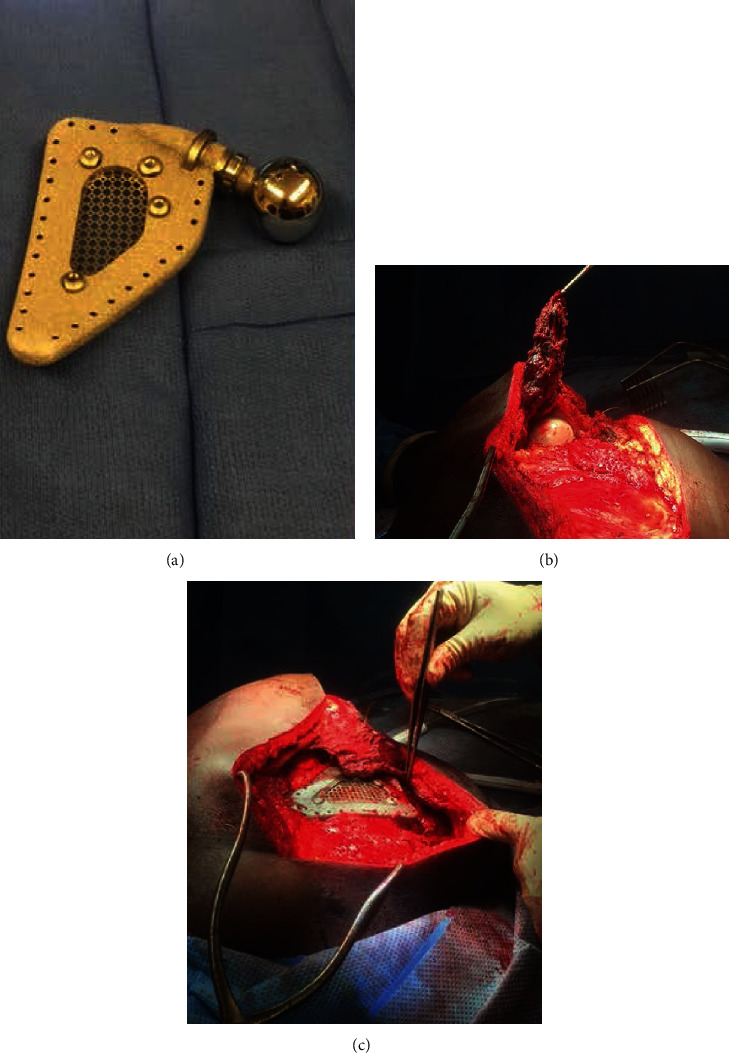
(a) Zimmer-Biomet custom total scapula reverse total shoulder prosthesis. (b) Isolation of the periscapular muscle flaps after excision of the scapula. (c) Incorporation of the soft tissue envelope into the scapular prosthesis through preexisting suture holes.

Attention is then focused on the humerus. Circumferential dissection is carried out to expose the humeral head and proximal humerus. The deltoid muscle is elevated from the acromion, taking care to maintain a cuff of normal tissue on the acromion for later repair. A large deltoid flap is elevated over the top of the humeral head, extending the flap well below the level of the rotator cuff insertion. An anterior capsulotomy is performed, and the rotator cuff tendons are reflected off of their insertion sites. An osteotomy is then performed at the anatomic neck of the humerus. The body of the scapula is then osteotomized, preserving the muscular insertions on the lateral, inferior, and medial borders (Figure 4(b)). The majority of the scapular body is then excised; this includes the superior portion of the body, the acromion, and the coracoid process. It is then possible to elevate the remaining osseous portion of the scapula, and subperiosteal dissection over the anterior aspect of the scapular body can be performed. Once wide en bloc resection of the tumor is completed, frozen sections of margins are reviewed.

Attention can then be focused on reconstruction, starting with the humerus. A reverse total shoulder proximal humerus component is placed in standard press-fit fashion. The final component that we use is a Zimmer-Biomet custom implant designed to receive the sphere on the custom total scapular prosthesis. This implant utilizes a retention-type glenoid and attached glenoid tray for increased stability. After the proximal humerus is reconstructed, the limb is held in anatomic resting position to act as a template for where the scapular prosthesis should be placed.

The scapular prosthesis is then placed and articulated with the glenoid tray on the proximal humerus (Figure 4(a)). If any viable cortical bone is left attached to periscapular musculature including the serratus anterior, levator scapula, rhomboids, and trapezius at the time of our radical resection, drill holes are made in the residual bony true scapular margins. #5 Fiberwire is passed through these holes and through associated holes in the scapular prosthesis to approximate the remnant native scapula and associated muscle tissue to the prosthesis, attempting to replicate anatomic positioning. This is performed circumferentially around the prosthesis, and the remaining free muscle flaps are then used to cover the posterior aspect of the prosthesis, and to reconstruct the rotator cuff (Figure 4(c)). Augmentation of the rotator cuff is performed with placement of an acellular dermal matrix allograft over the cuff tissues in 360-degree fashion. The graft is then sewn into the humerus itself, and passed through holes in the scapular prosthesis. The deltoid flap is then brought down and repaired with suture to the anterior shoulder, and into the prosthesis with existing suture holes, thus covering the entire shoulder girdle region and underlying construct. The subdermal and subcutaneous tissues are then approximated over a drain, completing the procedure.

## 4. Discussion

Historically, definitive treatment of large musculoskeletal malignancies about the shoulder girdle region involved forequarter amputation or flail extremity. The results of these procedures could be described as minimally functional and nonaesthetic. Before the 1970s, high-grade tumors about the shoulder girdle were almost always treated with forequarter amputation. It was not until the 1970s, when the Tickhoff-Linberg procedure was shown to achieve margins and survivorship similar to amputation, that limb salvage surgery was advocated for these tumors. This procedure classically involved en bloc resection of the scapula along with associated shoulder girdle musculature, as well as excision of the lateral clavicle and proximal humerus. Despite this advancement in treatment, patients were still not satisfied with their outcomes [7]. Griffin et al. reported the functional outcomes of patients undergoing total scapulectomy, including the glenohumeral joint, treated with a flail extremity. Seven out of eight patients ended up with a postoperative MSTS rating of poor [8].

Given the less than satisfactory outcomes with flail extremities, modifications to the classic Tickhoff-Lindberg procedure were drafted, including forms of humeral suspension. This typically involved stabilization of the humerus to the lateral clavicle with sutures or wires. Although stability was improved compared to a flail limb, marginal improvements with functionality or cosmesis were noticed. Despite these shortcomings, humeral suspension remained the most popular procedure after shoulder girdle resection until the late 1990s, when a scapular prosthesis was introduced. With a better understanding of endoprosthetic reconstruction, and the availability of custom-made components, improved outcomes have been reported after scapular reconstruction with prostheses [8, 9].

Pritsch et al. retrospectively compared outcomes of humeral suspension versus endoprosthetic reconstruction after total scapulectomy. In their reconstructions, they utilized a total scapula prosthesis from Howmedica Osteonics, combined with a proximal humerus prosthesis, and created an added constraint by reconstructing an artificial joint capsule out of Gore-Tex. They reported significantly improved functional and cosmetic results with reconstruction. They recommended that reconstructive efforts be undertaken when feasible [10].

Puchner et al. compared outcomes between patients treated with either the Tickhoff-Linberg procedure or endoprosthetic reconstruction after scapular resection. They used a custom-made scapula prosthesis from Biomet Orthopaedics coupled with a standard proximal humerus prosthesis. Their patients with endoprostheses reported good oncologic and functional outcomes, with MSTS scores far superior compared to patients who underwent the Tickhoff-Linberg procedure. They attribute their success to improved joint stability, which they believe leads to better function and strength below the level of the shoulder [7].

Mavrogenis et al. published a case report involving a patient with Ewing's sarcoma of the scapula treated with total scapulectomy and reconstruction with total scapula prosthesis and reverse-linked proximal humerus arthroplasty. Their patient was found to have a stable and painless shoulder 12 months postoperatively [11].

Savvidou et al. presented a series of 6 patients treated with total scapulectomy and reconstruction with a custom-made scapular prosthesis with constrained reverse total shoulder after malignant tumor resection. At final mean follow-up of 37 months, 2 patients had passed, leaving 4 patients who all had painless and stable shoulders, and satisfactory elbow, wrist, and hand function [12].

Tang et al. retrospectively reviewed ten patients who underwent total scapulectomy followed by reconstruction with constrained reverse total scapular prosthesis. The implant used was from ChunLi out of China and was comprised of a Co-Cr scapula with a reverse ball and socket hinge linking the proximal humeral prosthesis. At follow-up, they reported one dislocation, 1 infection, and a mean MSTS score of 76.7%. They concluded that constrained scapular prosthetic reconstruction after total scapulectomy for malignant tumors resulted in acceptable functional results and a low complication rate [13].

At our institution, a custom-made Zimmer-Biomet scapular prosthesis is created with a sphere at the reconstructed shoulder joint to accommodate a reverse total shoulder arthroplasty humeral prosthesis. Like any reverse total shoulder replacement, it is critical to evaluate for axillary nerve function and viable deltoid musculature. In the case of concurrent excision of malignancy about the shoulder girdle prior to reconstruction, care must be taken to protect the axillary nerve and preserve as much deltoid muscle as possible during dissection and resection of tumor, while at the same time being cognizant of tumor margins.

Malignant musculoskeletal tumors about the shoulder girdle region involving the scapula are fairly rare, but when diagnosed, challenging and complex surgical treatment may be warranted with the primary goal of improving patient survival. These tumors are typically large and extensive at the time of presentation, requiring radical resection to achieve margins and obtain local tumor control. Historically, forequarter amputation or flail extremity were the mainstays of treatment for this pathology. Presently, with advances in diagnostics, imaging, adjuvant therapies, and surgical treatment, it is reported that about 90% of patients presenting with malignant tumors involving the scapula are candidates for limb salvage surgery [14, 15].

We report on three cases of shoulder girdle sarcoma treated by a single surgeon (H.G.R.) with total scapular reverse total shoulder endoprosthetic reconstruction. Although complications were experienced, which can be expected at a high rate with radical reconstructive tumor surgery, we felt that this was acceptable in light of our functional and cosmetic outcomes, and patient satisfaction. However, we would recommend that such limb-sparing reconstructive procedures be performed only at specialized sarcoma centers by experienced surgeons with the sole focus on musculoskeletal oncology, in order to mitigate potential perioperative risks and complications.

## 5. Conclusion

Reconstruction with endoprosthesis seems to have gained acceptance as the preferred surgical treatment for extensive malignant musculoskeletal tumors about the shoulder girdle region involving the scapula, as this intervention has resulted in improved postoperative function and cosmesis, with an acceptable complication rate. After performing a literature review and reviewing the results of the three patients included in this case series, we conclude that reconstruction with total scapular reverse total shoulder prosthesis is a viable treatment and reconstructive option after radical excision of malignant lesions about the shoulder girdle region with extensive scapular involvement.

## Data Availability

The data used to support the findings of this study are reported in the article. Further information may be available from the corresponding author upon request.
